# Environmental Suitability of Kazakhstan to Highly Pathogenic Avian Influenza Using Data on Eurasian Outbreaks, 2020–2024

**DOI:** 10.3390/v17040574

**Published:** 2025-04-16

**Authors:** Asem Zh. Abenova, Yersyn Y. Mukhanbetkaliyev, Ablaikhan S. Kadyrov, Igor I. Sytnik, Alexander B. Shevtsov, Fedor I. Korennoy, Irene Iglesias Martin, Andres M. Perez, Sarsenbay K. Abdrakhmanov

**Affiliations:** 1 Faculty of Veterinary and Animal Husbandry Technology, S. Seifullin Kazakh Agro Technical Research University, Astana 010011, Kazakhstan; asem.abenova.1993@mail.ru (A.Z.A.); ersyn_1974@mail.ru (Y.Y.M.); kadyrov.ablaikhan@gmail.com (A.S.K.); 2 National Center for Biotechnology, Astana 010011, Kazakhstan; gu.nzmr@mail.ru (I.I.S.); ncbshevtsov@gmail.com (A.B.S.); 3 Federal Centre for Animal Health (FGBI ARRIAH), Vladimir 600901, Russia; korennoy@arriah.ru; 4 Federal Research Center for Virology and Microbiology, Branch in Nizhny Novgorod, Nizhny Novgorod 603950, Russia; 5 Centro de Investigación en Sanidad Animal (CISA), Instituto Nacional de Investigación y Tecnología Agraria y Alimentaria (INIA), Consejo Superior de Investigaciones Científicas (CSIC), 28130 Madrid, Spain; iglesias@inia.csic.es; 6 Center for Animal Health and Food Safety, College of Veterinary Medicine, University of Minnesota, St. Paul Campus, St. Paul, MN 55108, USA; aperez@umn.edu

**Keywords:** highly pathogenic avian influenza, outbreaks, epidemiology, maxent, Kazakhstan, suitability, underreporting

## Abstract

Highly pathogenic avian influenza (HPAI) is a highly contagious disease of domestic, synanthropic, and wild birds that has demonstrated a sharp rise globally since 2020. This study intends to examine environmental and demographic factors most significantly associated with HPAI (H5N1 and H5N8) outbreaks in Kazakhstan, 2020–2024, and to identify areas of potential underreporting of the disease. Two ecological niche models were developed, namely an “occurrence model” (considering climatic and environmental factors influencing the likelihood of HPAI occurrence) and a “reporting model” (that assesses the probability of disease reporting based on human and poultry population demography). Both models were trained using outbreak locations in countries neighboring Kazakhstan (Afghanistan, China, Hong Kong, Iran, Iraq, Pakistan and Russia), and then tested using the HPAI outbreak locations in Kazakhstan. Results suggested a good fit for both models to Kazakhstani outbreaks (test AUC = 0.894 vs. training AUC = 0.915 for “occurrence model”, and test AUC = 0.869 vs. training AUC = 0.872 for “reporting model”). A cluster of high occurrence-to-reporting ratio was detected in the south-western region of Kazakhstan, close to the Caspian Sea, suggesting a need for enhancing surveillance efforts in this zone as well as in some other areas of Pavlodar, Northern Kazakhstan, Western Kazakhstan, Qyzylorda, and Eastern Kazakhstan. Results presented here will help inform the design and implementation of control strategies for HPAI in Kazakhstan with the ultimate goal of promoting disease prevention and control in the country.

## 1. Introduction

Highly pathogenic avian influenza (HPAI) is a highly contagious disease of domestic, synanthropic, and wild birds, occurring as an epizootic or enzootic disease, characterized by damage to the respiratory organs and gastrointestinal tract, general depression, and decreased productivity [1]. HPAI cases are notifiable to the World Organization for Animal Health (WOAH), given its importance to animal health and its potential impact on public health [2]. Outbreaks caused by subtypes H5 and H7 of the HPAI virus are typically associated with severe clinical manifestations and high mortality, often leading to devastating consequences, with mortality rates that might be as high as 100% [3]. HPAI also represents a threat to public health due to its zoonotic potential, occasionally resulting in severe disease in humans. According to the World Health Organization (WHO), between 2003 and 2024, 954 cases of H5N1 HPAI with 464 human deaths have been registered in 24 countries [4].

Since 2020, an increasing number of HPAI outbreaks have been reported worldwide [2,5,6,7,8]. A total number of 26,674 HPAI outbreaks were reported between 2021 and 2024 worldwide, ranging from 1819 in 2021 up to 9148 in 2023 [9]. Due to the movement of infected wild birds and international trade [10,11], HPAI is recognized as a transboundary animal disease. For example, in 2020, while an HPAI epidemic was ongoing in Kazakhstan, outbreaks were also recorded in China, Russia, Iraq, and Vietnam [9,12,13,14,15]. At the time when this manuscript was written in April 2025, outbreaks are still ongoing in the poultry population of Afghanistan, China, India, Korea, the Philippines, and Vietnam (subtypes H5, H5N1, H5N2, H5N5, H5N6 and H7N9) [4,9,13,16,17].

The first HPAI (H5N1) outbreaks in Kazakhstan were registered in 2005, and a “stamping-out” strategy was applied to control the disease nationwide [18,19]. In 2007, an intensive vaccination program of poultry was implemented using an inactivated emulsified vaccine against avian influenza in chickens, geese, ducks and turkeys, produced by the Research Institute for Biological Safety Problems of the Ministry of Healthcare, Republic of Kazakhstan. Despite these measures, in 2020, HPAI caused substantial damage to the poultry industry of Kazakhstan. In September 2020, the country’s Ministry of Agriculture reported an HPAI outbreak in Northern Kazakhstan that spread into other regions of the country. A total of 11 outbreaks were registered that year in Akmola, Almaty, Kostanay, Pavlodar and Northern Kazakhstan [20]. In 2021, five additional outbreaks were reported in the Akmola, Aktobe, Pavlodar, and Northern Kazakhstan regions [5,9,21]. In the outbreaks, quarantine was declared, and temporary restrictions were introduced on the export of live poultry and poultry products, which had a significant impact on the rise in prices for eggs and poultry meat [22].

Several seasonal bird migration routes pass through the territory of Kazakhstan. Most importantly, the Central Asian–Indian and East Asian bird migration routes intersect with the Black Sea–Mediterranean and East African–West Asian pathways in the western part of the country [23]. As a consequence of such diversity, 433 species of wild birds have been registered during the nesting, molting, seasonal migrations, and wintering periods [24]. Every year, the number of nesting bird species is believed to reach 10 million, 2–3 million birds arrive for molting, and about 50 million migratory birds stop on Kazakhstani water bodies during the spring and autumn migrations [25]. The whole-genome sequencing of avian influenza virus identified in Kazakhstan in 2020 showed that the strains were associated with those from Southern Russia, the Caucasus, the Ural region, Southwestern Siberia and Eastern Europe [5]. The influenza virus A/chicken/North Kazakhstan/184/2020 (H5N8) isolated during an outbreak in Northern Kazakhstan has shown great genetic similarity with HPAI H5N1 viruses of poultry from the Middle East and West Africa [21]. Also, during the influenza outbreaks reported in 2021 in poultry in the Northern Kazakhstan and Akmola regions were caused by the avian influenza A/H5N8 virus belonging to the 2.3.4.4b clade with a high level of homology (98.42–98.70%) to the strains from China [22]. In addition, during the epidemiological surveillance of avian influenza viruses in wild birds in 2018–2019, the simultaneous circulation of genome segments of Asian, European, and Australian genetic lineages of avian influenza H3N8 virus was established in wild birds, which confirms the important role of Kazakhstan and Central Asia as a center for the transmission of avian influenza viruses, linking the migration routes from East Asia to Europe and vice versa [25,26,27].

Despite the long history of studying the ecology of avian influenza viruses in Kazakhstan, many key aspects of the HPAI epidemiology remain insufficiently understood. The purpose of this work was to identify areas of Kazakhstan likely to be infected but unreported. Towards that goal, the suitability of the territory to the occurrence of HPAI outbreaks was modeled using an ecological niche model based on information from neighboring countries to assess the impact of climatic, landscape and socio-economic factors on the probability of the disease occurrence and reporting separately. Predictions were subsequently overlapped to identify clusters of regions with high probability of occurrence but low probability of reporting. Results will help evaluate the effectiveness of the surveillance system for HPAI in Kazakhstan, contributing to the implementation of measures intended to prevent and mitigate the impact of the disease in the country.

## 2. Materials and Methods

### 2.1. Study Area

**Figure 1:**
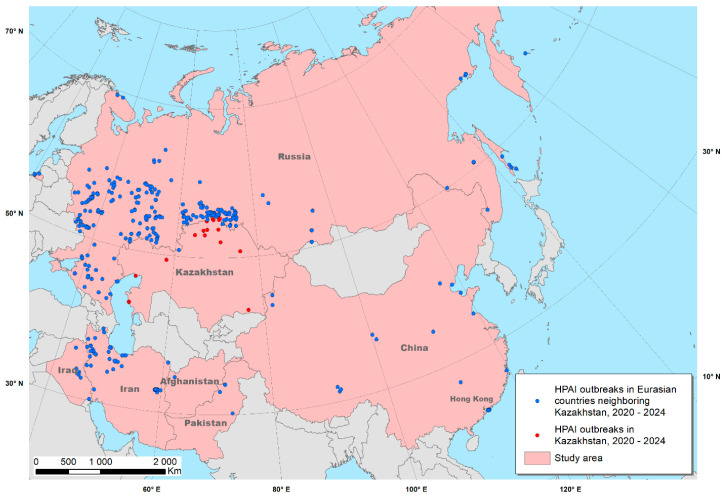
Geographical location of highly pathogenic avian influenza (HPAI) outbreaks reported in Kazakhstan and neighboring countries between 2020 and 2024.

The study area comprised Kazakhstan and neighboring countries in Eurasia, considered to be relatively similar to Kazakhstan in terms of their geographic location, and in which HPAI outbreaks were reported in 2020–2024. Those countries included Afghanistan, China and Hong Kong, Iran, Iraq, Pakistan and Russia (Figure 1). Because few outbreaks were reported in Kazakhstan, data from these countries were necessary to identify associations between outbreaks and predictors contributing to the ultimate objective of identifying areas in which the disease may have been underreported in Kazakhstan.

### 2.2. HPAI Data

Spatially referenced data on HPAI outbreaks were collected from the Food and Agriculture Organization (FAO) Empres-i database (https://empres-i.apps.fao.org/(accessed on 24 February 2025)). The attributes of the data include geographical coordinates, virus subtype, location name, disease observation and reporting dates, and source of information. Only outbreaks reported to WOAH were used for the analysis. The total number of HPAI outbreaks during the study period was 17 in Kazakhstan (hereinafter “test data”) and 444 in the rest of the study area (hereinafter “training data”) (Table 1). Outbreaks in domestic and wild birds were treated together as indicators of HPAI virus presence [28].

### 2.3. Study Design

Two types of suitability models were developed. The first model, fitted using environmental factors as predictors, assumes suitable conditions for the disease occurrence itself (hereinafter “occurrence model”). The second model, including only factors related to the presence of human and poultry populations, assumes the probability of reporting HPAI outbreaks (hereinafter “reporting model”).

First, both models were built for the whole study area using only training data. Subsequently, the models were tested on Kazakhstan data to evaluate whether the trained models were able to adequately predict the observed HPAI outbreaks in Kazakhstan. Finally, both models were compared by estimating a pixel-by-pixel “occurrence-to-reporting” ratio in order to reveal areas where potential underreporting might have taken place. Additionally, the number of HPAI outbreaks by country was compared to the summary environmental/demographic suitability in the corresponding country, calculated as the sum of cells with suitability >50%.

### 2.4. Ecological Niche Models

An ecological niche model based on the principle of Maximum Entropy (Maxent) was used to evaluate both “occurrence” and “reporting” suitability to HPAI [29]. We chose a set of potential explanatory variables, including a number of climatic, landscape and socioeconomic factors (Table 2), which demonstrated significance in explaining the observed distribution of HPAI cases in similar studies [28,30,31,32]. The set of variables included population density, land cover, human footprint index, distance to water bodies, chicken population density, maximum green vegetation fraction, altitude, mean yearly air temperature, yearly precipitation, minimum temperature of coldest month, precipitation seasonality, precipitation of driest quarter, and precipitation of warmest quarter. All raster layers were clipped by the extent of the study area and transformed to a 10 × 10 km^2^ resolution, defined by the layer with the coarsest resolution (namely, the FAO global chicken density). Model performance was assessed by the mean AUC (Area Under the Curve indicates an area under a receiver operating characteristic (ROC) curve, which evaluates the predictive accuracy of a Maxent model) value that indicates the ability of the model to correctly predict any random presence location. AUC values exceeding 0.8 were considered a “high indicator”, values 0.7 < AUC < 0.8 were considered “good indicator”, whereas AUC < 0.7 indicated no predictive power of the model [33].

To build an “occurrence model”, we used only climatic and environmental factors (Table 2), which presumably shape suitable conditions for HPAI outbreak emergence. For the “reporting model”, we only used population-related factors (Table 2), which may indicate the presence of birds and the probability of observing and reporting outbreaks by humans.

Further, test data (namely, HPAI outbreaks in Kazakhstan) were used in the same models to assess the models’ performance. The test AUC values were obtained and compared with those produced by the trained model.

To test the importance of the variables, a Jackknife test was used. For each individual variable, the model was alternatively run with and without the given variable, comparing the predictive gain associated with the addition of the variable.

To compare predictions of both the “occurrence” and “reporting” models, we calculated their pixel-by-pixel ratio, which indicates whether a probability of HPAI occurrence was higher or lower than the probability of its reporting. Further, clusters of high values were identified using the normal model of the spatial scan statistic. Briefly, this model identified spatial clusters in which the proportion of cells in which the occurrence-to-reported ratio was significantly higher (*p* < 0.05) than the expected under the null hypothesis of even distribution of the ratio in Kazakhstan [34].

To assess potential over- or underreporting of HPAI outbreaks by country, we calculated a summary suitability (derived from both “occurrence” and “reporting” models) as the number of raster cells per country in which the predicted suitability exceeds 50%. We then applied a Negative Binomial linear regression model to assess the goodness-of-fit of the summary suitability as a predictor of the number of outbreaks per country.

### 2.5. Software

Spatial data mapping and processing were performed using ArcGIS for Desktop v 10.8.2 (Esri, Redlands, CA, USA). The maximum entropy model was fitted using Maxent software version 3.4.4 [35]. The negative Binomial model was built using the MASS package, implemented in the statistically oriented programming software R version 4.2.1 [36]. The normal model of the spatial scan statistic was performed in the SatScan v 10.1 software [37].

## 3. Results

**Figure 2:**
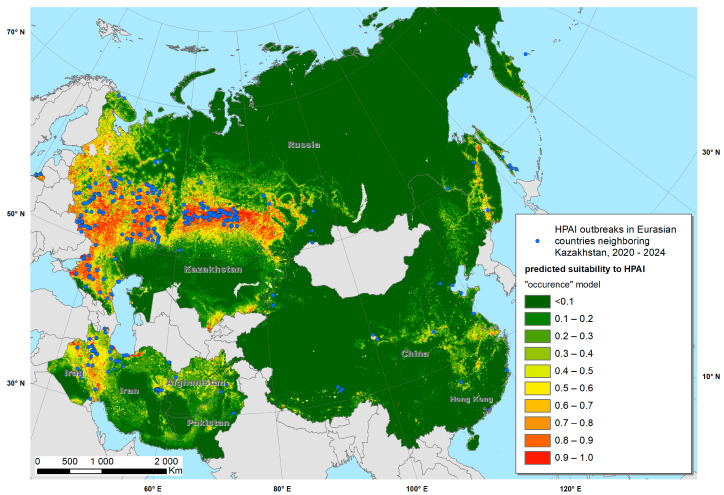
Predicted suitability of Kazakhstan and neighboring countries to highly pathogenic avian influenza (HPAI) occurrence fitted using a Maxent model.

**Figure 3:**
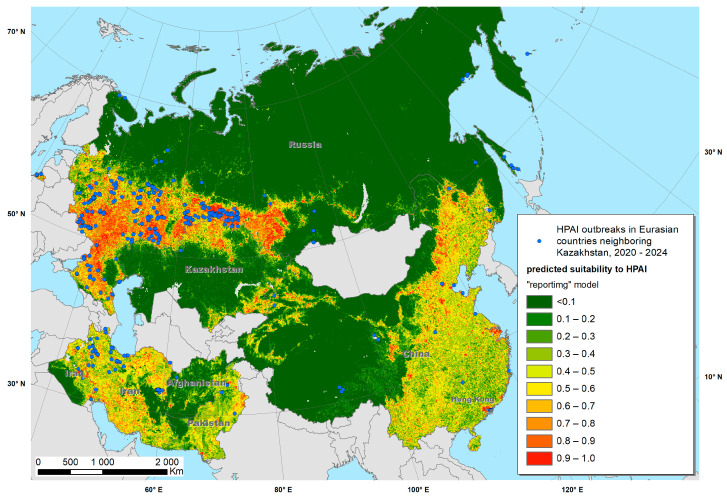
Predicted suitability of Kazakhstan and neighboring countries to highly pathogenic avian influenza (HPAI) reporting, fitted using a Maxent model.

**Figure 4:**
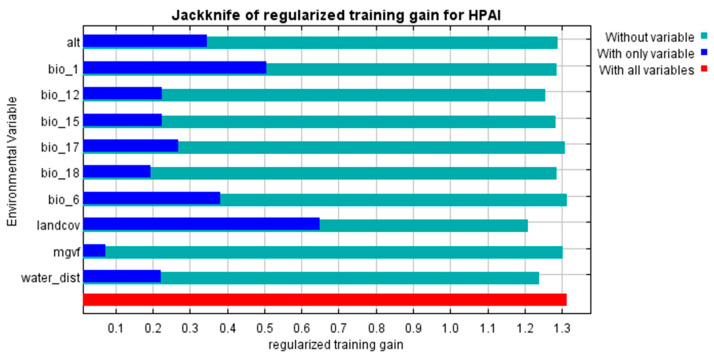
Results of a Maxent Jackknife test to assess the relative contribution of variables used to predict the occurrence of highly pathogenic avian influenza (HPAI) in Kazakhstan.

**Figure 5:**
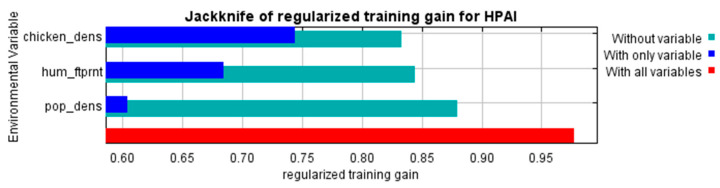
Results of a Maxent Jackknife test to assess the relative contribution of variables used to predict the reporting of highly pathogenic avian influenza (HPAI) in Kazakhstan.

The Maxent models created with the training data provided predictive suitability maps for “occurrence” (Figure 2) and “reporting” (Figure 3) and demonstrated good performance, with a mean AUC of 0.915 and 0.872, respectively. The variables that contributed most to the “occurrence model” were land cover type (specifically water, arable land, urban and built-up areas, mixed arable land and natural vegetation categories), mean annual air temperature, annual precipitation, mean temperature of coldest month and distance to water bodies (Figure 4 and Figure 5). The “reporting model” demonstrated chicken population density and human footprint index to be the most significant predictors.

Not surprisingly, when applied to outbreaks in Kazakhstan (test data), both models demonstrated a slightly lower AUC of 0.894 and 0.869 for “occurrence” and “reporting”, respectively, compared to the values reported for the models used to assess neighboring countries.

**Figure 6:**
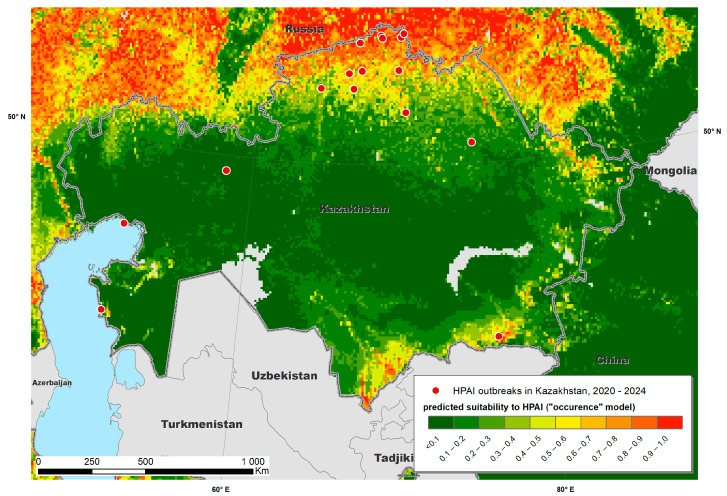
Predicted suitability of Kazakhstan to the occurrence of highly pathogenic avian influenza (HPAI) estimated using a Maxent model.

**Figure 7:**
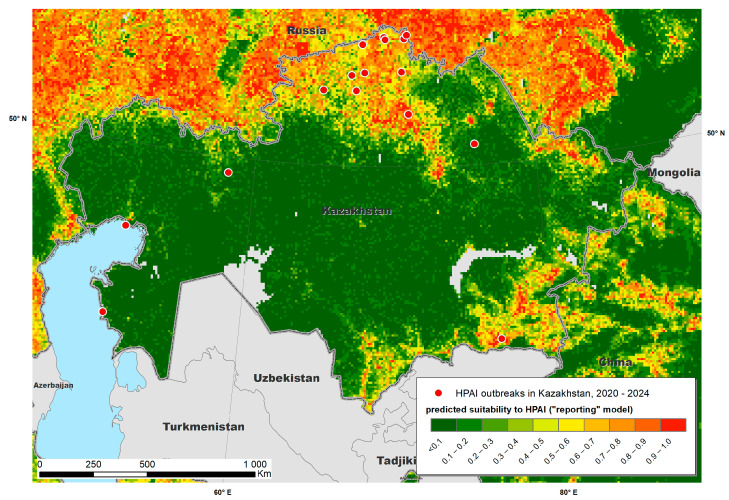
Predicted suitability of Kazakhstan to the reporting of highly pathogenic avian influenza (HPAI) estimated using a Maxent model.

A map of predicted environmental suitability for Kazakhstan (“occurrence model”) overlaid with test data is presented in Figure 6, whereas the “reporting model” output is presented in Figure 7. Both maps suggested that most HPAI outbreaks in Kazakhstan were recorded in areas predicted by the Maxent model as “suitable”, which confirms the adequacy of the model and underlines the possibility of further improvement in the model by adjusting the set of environmental variables.

**Figure 8:**
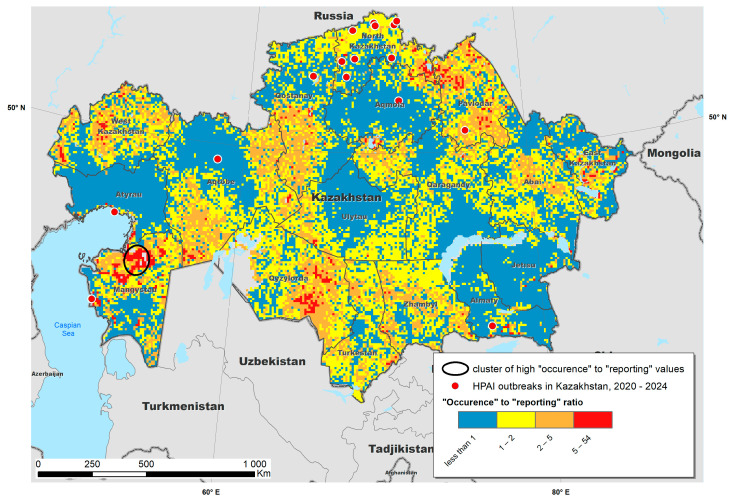
Occurrence-to-reporting ratio estimated for highly pathogenic avian influenza (HPAI) estimated in Kazakhstan. The black oval indicates the location of a cluster in which the ratio was significantly higher than expected.

The “occurrence-to-reporting” ratio, along with the location in which the ratio was higher than expected, is provided in Figure 8. The cluster was located in the south-western part of Kazakhstan, close to the Caspian Sea. Other areas in which environmental suitability significantly exceeded the population-based reporting probability included large parts of Pavlodar, Northern Kazakhstan, Western Kazakhstan, Qyzylorda and Eastern Kazakhstan regions, suggesting a potential underreporting in those areas.

The summary suitability against the reported number of outbreaks by country is presented in Table 3. The regression model revealed a high significance of the summary “occurrence” suitability as a predictor of the number of outbreaks with *p* < 0.001 and R^2^ = 0.71, while the performance of the “reporting” model was slightly lower (*p* < 0.001, R^2^ = 0.68).

## 4. Discussion

This study contributed to the elucidation of the most general patterns on the suitability of Kazakhstan to HPAI outbreaks and the identification of areas of potential underreporting in the country. With that objective, two models were compared, the first of which was based on the environmental factors shaping favorable conditions for HPAI occurrence, and the second on demographic factors influencing the probability of disease reporting. A similar, although not identical, approach was followed by Sun et al. [28]. Our modeling demonstrated a good fit of both models and allowed the revealing of some general factors contributing to the observed pattern of HPAI distribution in Kazakhstan. In the occurrence model, land cover demonstrated the strongest relationship with HPAI outbreaks, followed by annual air temperature, precipitation and air temperature of the coldest month. The distance to water bodies was also a significant factor, presumably due to the role of migratory wild birds, often implicated as potential hosts of HPAI [38]. For the most influential variable, namely, chicken density, a positive relationship was found with HPAI suitability, which indicates a naturally expected relationship between the presence of a highly dense susceptible population and disease transmission. A similar dependency was found for the human footprint index. A stronger human impact indicates higher exposure to HPAI (presumably due to the most intensive between-farm contact as well as increased probability of the disease detection), which is in line with findings of Li et al. [39].

When we compared summary suitability (proportion of highly suitable cells) against actual reported outbreaks (2020–2024) across the study area, Kazakhstan generally aligned with patterns observed in other countries in the dataset.

The ratio between predictions obtained from both models at any given location may suggest areas in which the probability of disease occurrence was significantly higher than the probability of its reporting, compared to the background probability of the country. Such areas may be treated as potential areas of underreporting and require enhanced monitoring measures. In addition to the Mangystau region, indicating the most significant concentration of high “occurrence to reporting” cells, areas of elevated values were also detected throughout Kazakhstan. In particular, large areas of the Qyzylorda, Turkestan, Zhambyl regions in the south, Abai, Pavlodar, North Kazakhstan in the north, as well as the North Kazakhstan and West Kazakhstan regions, demonstrated large territories investigated by our modeling as places of potential underreporting.

The Caspian Sea is located on the migration routes of wild birds, and it is part of the Central Asian Flyway. This route covers vast areas from Siberia to South Asia, including the western regions of Kazakhstan. Migratory birds such as ducks, geese, swans, raptors and other species such as flamingos actively follow the wetlands and coastal areas of the Caspian Sea for stopovers, nesting and wintering [38,40]. Due to the diversity of ecosystems in the Caspian Basin, this region provides important resources for maintaining the biological diversity of migratory species. Of particular importance are the unique ecosystems of the Ural (Zhaiyk) River delta and the northern coast of the Caspian Sea, where vast wetlands are formed. These areas serve not only as transit points for millions of migratory birds but also as concentration sites for rare and endangered species. Most importantly, the western regions of Kazakhstan (Atyrau, Mangistau and West Kazakhstan) are characterized by active development of the poultry industry. Proximity of poultry farms to migration routes increases the likelihood of contact between domestic and wild birds, which increases the risk of HPAI transmission [41,42].

Interestingly, HPAI outbreaks reported in Kazakhstan were mainly located in high-risk areas, predicted by both models, suggesting that the models adequately capture the pattern of HPAI environmental and demographic dependence. This observation suggests that, even in the presence of underreporting, affected regions were properly identified, facilitating the design and implementation of actions intended to contain disease spread.

An important limitation of this study was that data from Kazakhstan were limited. The inclusion of data from other countries in the region represented an attempt to mitigate the impact of this limitation, at least in part. Nevertheless, it is uncertain how effective this strategy may have been in adjusting for potential reporting bias. Additionally, the reliance on officially reported outbreaks introduces the possibility of underreporting. Remote or less-monitored regions may lack resources or infrastructure to detect and confirm infections, an issue raised in multiple HPAI modeling studies, where more thorough reporting in certain areas (e.g., parts of Europe with robust wildlife case reporting) can lead to overrepresentation of alerts [43]. Our analysis indicates that the areas we flagged for potential underreporting could, in reality, have an even higher burden of unnoticed HPAI activity.

Although migratory birds are emphasized here as vectors for transboundary movement, the poultry trade remains a significant contributor to HPAI spread [44]. Indeed, advanced molecular epidemiological approaches can help delineate how much of a particular outbreak cluster originates from wild bird introductions versus the human-mediated poultry trade. Integrating whole-genome sequencing data would improve our risk maps and inform more targeted control strategies.

In summary, the results here contributed to identifying three areas of high risk for HPAI cases in Kazakhstan, associated with the presence of wild and domestic birds. Strengthening early detection systems in these high-risk areas, such as expanding active surveillance in wetlands or developing and implementing a real-time early warning system [45], could enhance the ability to anticipate viral incursions. This proactive approach would provide a critical time advantage for outbreak control by selectively triggering active sampling and response measures, ultimately contributing to the prevention of further disease dissemination within the country.

## Figures and Tables

**Table 1:** Number of highly pathogenic avian influenza (HPAI) outbreaks reported in Kazakhstan and neighboring countries between 2020 and 2024.

Number of HPAI Outbreaks	In Domestic Birds	In Wild Birds
Afghanistan	1	0
China	1	13
Hong Kong	0	12
Iran	57	5
Iraq	8	0
Kazakhstan	14	3
Pakistan	5	0
Russia	241	101

**Table 2:** List of environmental variables used to fit an ecological niche modeling of highly pathogenic avian influenza (HPAI) in Kazakhstan and neighboring countries.

Variable Name	Variable Meaning	Factor Type	Measurement Unit
Alt	Altitude above sea level	environmental	meters
landcov	Type of land cover	environmental	categories
Mgvf	Maximum green vegetation fraction	environmental	%
pop_dens	Population density	population	persons/km^2^
water_dist	Distance to water bodies	environmental	kilometers
hum_ftprnt	Human footprint index (a measure of human influence on the terrestrial systems of the Earth)	population	index
chicken_dens	Chicken density	population	head/km^2^
bio_1	Annual mean air temperature	climatic	°C × 10
bio_6	Minimum air temperature of the coldest month	climatic	°C × 10
bio_12	Annual precipitation	climatic	millimeters
bio_15	Precipitation seasonality	climatic	%
bio_17	Precipitation of the driest quarter	climatic	millimeters
bio_18	Precipitation of the warmest quarter	climatic	Millimeters

**Table 3:** Summary suitability by country and reported number of highly pathogenic avian influenza (HPAI) outbreaks in Kazakhstan and neighboring countries.

Country	Summary “Occurrence” Suitability	Summary “Reporting” Suitability	Number of HPAI Outbreaks Reported
Afghanistan	180	267	1
China	671	17,840	14
Hong Kong	1	2	12
Iran	2024	6891	62
Iraq	718	1054	8
Kazakhstan	2069	2970	17
Pakistan	108	1745	5
Russia	23,653	22,174	342

## Data Availability

The data used in the present research (specifically, HPAI outbreaks location data and environmental layers) are publicly available, and their sources are listed.
